# Effectiveness of a skin care programme for the prevention of contact dermatitis in healthcare workers (the Healthy Hands Project): A single‐centre, cluster randomized controlled trial

**DOI:** 10.1111/cod.13214

**Published:** 2019-03-15

**Authors:** Maryam Soltanipoor, Sanja Kezic, Judith K. Sluiter, Fleur de Wit, Angela L. Bosma, Ruth van Asperen, Thomas Rustemeyer

**Affiliations:** ^1^ Coronel Institute of Occupational Health, Amsterdam Public Health Research Institute Amsterdam The Netherlands; ^2^ Department of Dermatology Amsterdam UMC (De Boelelaan) Amsterdam The Netherlands; ^3^ Department of Dermatology Amsterdam UMC (Meibergdreef) Amsterdam The Netherlands

**Keywords:** hand dermatitis, hand moisturizers, healthcare, nurses, prevention

## Abstract

**Background:**

Healthcare workers (HCWs) are at risk of developing hand dermatitis (HD). Guidelines recommend moisturizers to prevent HD, but in practice their effectiveness has been poorly investigated.

**Objectives:**

To assess whether an intervention aimed at improving skin care leads to a reduction in HD severity.

**Methods:**

In this 1‐year randomized controlled trial, 9 wards (285 HCWs) were allocated to an intervention group (IG), and 10 wards (216 HCWs) were allocated to the control group (CG). The intervention included provision of cream dispensers with electronic monitoring of use, regularly communicated to the HCWs. The primary and secondary outcomes were change from baseline in Hand Eczema Severity Index (HECSI) score (ΔHECSI) and change in natural moisturizing factor (NMF) level (ΔNMF).

**Results:**

At 12 months, the rates of loss to follow‐up were 41% and 39% in the IG and the CG, respectively. The HECSI score was reduced in the IG by −6.2 points (95%CI: −7.7 to −4.7) and in the CG by −4.2 points (95%CI: −6.0 to −2.4). There was no significant difference in ΔHECSI or ΔNMF between the groups. Relative improvement in the HECSI score was significantly higher in the IG than in the CG (56% vs 44%). In a subgroup of HCWs with mild HD, the IG showed a larger HECSI score decrease than the CG (*P* < 0.001).

**Conclusion:**

Although there was no significant effect on the primary outcomes, the intervention showed overall positive effects on the HECSI score.

## 1 INTRODUCTION

Occupational skin diseases (OSDs), mainly comprising irritant contact dermatitis (ICD), are common work‐related diseases, mostly localized on the hands.^1^ OSD impacts on the quality of life of affected workers, and poses a serious threat to ability to work ability as well as a high socio‐economic burden. In The Netherlands, the annual costs of medical care, sick‐leave and disability pensions attributable to OSD in 2011 have been estimated to be €98 million.^2^ Continuous exposure to “wet work” and its deleterious effect on the skin barrier has been identified as a major risk factor for OSD, in particular ICD on the hands. One of the most affected populations comprises healthcare workers (HCWs), with an estimated point prevalence of 12% to 30%.^3^, ^4^ The risk of occupational hand dermatitis (HD) in the health sector is increasing, as hand hygiene for infection control has become more rigorous, and the use of hand sanitizers and hand washing has been intensified to prevent infections.^5^ Apart from the adverse effects on the quality of life of affected HCWs, HD can negatively influence the safety of the patients. Recent studies reported that HCWs with HD avoid the use of hand disinfectants, because of a stinging sensation when disinfectants are used on damaged skin, and a belief that disinfectants will further aggravate the symptoms.^6^ Obviously, prevention of HD in the healthcare sector is critical for both HCWs and patients. In several countries, national guidelines for the prevention of OSD have been established, following a common hierarchical structure in the prevention of occupational diseases. First, hazards have to be eliminated or exposure reduced by the use of technical and organizational measures. When this is not sufficient, personal protection and behavioural measures have to be adopted. However, in the healthcare sector, elimination or reduction of the hazards is difficult, owing to hygiene requirements.^7^ Replacement of the main risk factor, hand washing, with hand disinfectants has been proposed, but hand washing cannot be completely avoided, as it is strictly prescribed by some hygiene protocols.^8^ As skin barrier damage is the aetiological factor in ICD, several guidelines emphasize the importance of the maintenance of a competent skin barrier in its prevention. Thus, the guidelines of the Netherlands Society of Occupational Medicine recommend the use of emollients to maintain a competent skin barrier.^9^ In recent guidelines of the ESCD, application of moisturizers to the hands is recommended during the working day, but especially after work and before bedtime.^10^

Although experimental studies have shown skin barrier‐enhancing effects of emollients in ICD patients,^11^ there are very few studies on their efficacy in occupational settings, and the effect of emollients has mainly been studied as a part of multifaceted interventions.^12^, ^13^ The use of emollients in the workplace, however, remains low in general.^14^

This study was therefore focused on improving hand cream use by HCWs to prevent HD symptoms. The intervention strategy was inspired by experience from hand hygiene studies showing positive effects of continuous monitoring and feedback in improving compliance.^15^ Feedback, in particular, has been suggested to be effective in improving performance when: (a) baseline performance is low; (b) when it is provided by a supervisor; (c) when it is provided more than once; and (d) when it is provided both verbally and in written form.^16^ Group monitoring is widely recognized as being more effective than other monitoring systems that track individuals’ actions, which can be seen by staff as punitive or an intrusion of their privacy.^17^ Recently, we reported on an electronic monitoring system developed for the continuous registration of hand cream consumption with electronic dispensers in wards.^18^ This system enables detailed feedback on the frequency and time‐pattern of hand cream use on wards. In the present study, we aimed to assess the effectiveness of this system in a randomized controlled trial (RCT). The intervention comprised installing hand cream dispensers on the wards, electronic monitoring of use, and repeated feedback. The effect of the intervention was measured as the change in clinical severity of HD between baseline and follow‐up at 12 months, as compared with the control group (CG). Furthermore, as a secondary outcome, we determined the change in the stratum corneum (SC) levels of natural moisturizing factors (NMFs) as a potential sensitive biomarker of skin barrier damage. Cluster randomization was used to minimize the threat of “treatment contamination.” This article is focused on the primary and secondary outcomes of the intervention. We will report the effects of the intervention on cream use behaviour in a separate article.

## 2 PATIENTS AND METHODS

The Healthy Hands Project is a single‐centre, cluster‐randomized, parallel‐group controlled trial with blinded outcome assessments. We have previously described the design^18^ and statistical methods^19^ in detail.

The participants in this study were HCWs clustered in wards of the Amsterdam University Medical Centre, Amsterdam, The Netherlands. They were enrolled between 2 May and 14 June 2016. Only wards known to have substantial exposure to wet work were included. These wards were categorized as low‐exposure or high‐exposure wards on the basis of soap purchase in the previous year. The eligibility criteria were provision of written informed consent, being employed as an HCW or nutrition assistant on one of the wards, and being exposed to “wet work.” Ethics approval to conduct the trial was granted by the Medical Ethics Review Board of the Academic Medical Centre (AMC) (reference number, NL54372.018.15; Trial registration, NTR 5564; date of registration, November 2, 2015). Informed consent to participate in the trial was obtained from all participants.

### 2.1 Intervention

The intervention comprised provision of hand cream dispensers placed at accessible locations in the wards, continuous electronic monitoring of cream use, and feedback on cream use at ward level. The feedback was provided by means of posters reflecting the compliance of HCWs with the skin care recommendations that they were given. The HCWs were instructed to use hand cream at least two times per shift, preferably before starting a shift, following wet work activities, or after finishing a shift. The hand cream in the electronic dispensers, Stokoderma Aqua Sensitive from the Stoko‐Deb Group, was a perfume‐free and silicone‐free soft cream, with no particular pharmacological function. The main ingredients were moisturizing compounds, such as glycerol and urea.^18^ Both the intervention group (IG) and the CG received basic education on skin care and skin protection behaviour every 3 months from baseline to the end of the study. This education took the form of a short small‐group lesson lasting 5 to 10 minutes. The objective was to assess the effectiveness of the intervention in reducing the severity of HD in HCWs exposed to wet work. The primary outcome was the difference in the Hand Eczema Severity Index (HECSI) score between baseline and 12‐month follow‐up (ΔHECSI). The secondary outcome was the difference in NMF levels determined in the SC samples collected at baseline and follow‐up (ΔNMF).

### 2.2 Assessment

At baseline, HCWs completed a questionnaire on self‐reported atopic dermatitis, here defined as a history of flexural dermatitis on the elbows or knees, a history of HD, glove use, frequency of hand washing, and the use of hand alcohol and hand creams at work. All outcomes were evaluated at the individual level.

The clinical assessment of the hands was performed by a trained physician (M.S.) using the HECSI scoring system.^20^ The HECSI grades the intensity (scale 0‐3) of clinical symptoms, including erythema, vesicles, fissures, scaling, papules and oedema in combination with the extent (affected area) of disease (scale 0‐4) per area of the hand (fingertips, fingers, palms, backs of hands, and wrists). To obtain the total score, the extent per location was multiplied by the intensity of all clinical symptoms and summed.^20^ The HECSI score ranges from 0 (no dermatitis) to 360 (extremely severe dermatitis). The severity of disease was classified as mild (0‐11 HECSI points), moderate (12‐27 points) and severe (≥28 points) dermatitis.^21^ The minimal detectable change (MDC) in the HECSI score was defined according to Norman et al^22^ as 0.5 times the standard deviation of the mean ΔHECSI value.^22^ The MDC represents the smallest change that can be detected by a measurement instrument that corresponds to a true change rather than being attributable to measurement error.

### 2.3 NMF analysis

The SC samples for the NMF analysis were collected with a tape‐stripping technique. Round, adhesive tapes (3.8 cm^2^, D‐Squame; CuDerm, Dallas, Texas) were attached to the dorsal part of the dominant hand, and pressed on for 5 seconds with a standardized force (D500, D‐Squame Pressure Instrument; CuDerm). The tape stripping from the same skin site was repeated with a new tape five consecutive times. For the NMF analysis, the fifth consecutive tape was used.^18^, ^23^ For the analysis, a slightly modified method of Dapic et al was applied.^24^ Briefly, NMF was extracted from the tapes by two successive extractions with 0.5 mL of water. The concentration of NMF in the extracts was analysed with high‐performance liquid chromatography with ultraviolet detection.^24^ In contrast to the published protocol, the protein levels on the tape used to compensate for variable amounts of the SC being removed by a tape were not estimated from the optical density values, because visual inspection of the tapes showed that the SC was not evenly distributed over the tape, which is the prerequisite for applying this method. Instead, the protein amount on the tape was determined in the extracts with a Pierce bovine serum albumin assay (Thermo Fisher Scientific, Rockford, Illinois).^24^ The NMF levels were expressed as mmol/g protein.

### 2.4 Randomization and statistical analysis

We based the sample size calculation on the Osnabruck Hand Eczema Severity Index score,^25^ as there were no studies available on primary prevention in which the HECSI was used, and the two scores are well correlated (*r* = 0.85; *P* < 0.001). Using a two‐sided *t* test with a significance level of 0.05, we calculated that a study with 17 wards per treatment group with 16 HCWs per ward (a total of 544 HCWs) would have 81% power to detect a difference of 0.4 in group means,^25^ on the assumption of an SD of 1.2 points and an intracluster correlation of 0.05.^18^ These power calculations were performed in pass 15 (NCSS, Kaysville, Utah).

The HCWs were randomized to the IG or the CG at the ward level. Wards (as the unit of randomization) were randomized in fixed size blocks of two, and stratified into “high” or “low” levels of exposure to “wet work.” Wet work exposure was estimated at the ward level from the quantity of soap purchased in the period January to May 2016. The first half of the wards with the highest soap purchase were categorized as high‐exposure, and the lower half as low‐exposure. The wards were randomized by use of a secure Internet‐based service (www.sealedenvelope.com). The randomization sequence was generated by the principal investigator (S.K.), who was not involved in enrolling HCWs or assessing outcome measurements. The researcher (M.S.) who performed the outcome measurements and statistical analysis was blinded to treatment allocation until all data had been collected and cleaned, and a statistical analysis plan had been published. The HCWs were not blinded to treatment allocation.

The statistical analysis plan has been described in detail previously.^19^ Briefly, characteristics of wards, working years, working hours, sex, self‐reported atopic dermatitis, self‐reported HD in the past year and baseline NMF and HECSI values are presented by the use of descriptive statistics, and no formal statistical testing was performed. We used counts and percentages to present categorical variables. For continuous variables, the mean and SD (normally distributed data) or the median and interquartile ranges (IQRs) (deviation from a normal distribution) were used. The intraclass correlation (ICC) was also calculated. Two‐sided *P* values of <0.05 were considered to be statistically significant. Statistical uncertainty was expressed by the use of two‐sided 95% confidence intervals (CIs). The analyses were performed by an investigator (M.S.) supervised by the other investigators (S.K. and J.S.) and the Clinical Research Unit of the AMC. All statistical analysis was performed with ibm spss statistics version 24 (IBM Corp., Armonk, New York). When writing this manuscript, we adhered to the CONSORT statement.^26^, ^27^

The main analyses of the primary and secondary outcomes was performed on a modified ‘intention‐to‐treat’ population, consisting of all participants with a HECSI score at baseline and 12 months (i.e. per protocol population). We present crude means and 95%CIs for ΔHECSI and ΔNMF for the IG and the CG. To model the absence of ΔHECSI values when HECSI scores at 12 months were not available, we used a simple joint model approach. We used a linear model for ΔHECSI and ΔNMF, and a binary model with a logit link function for the missing data. We obtained *P* values for the difference between the IG and the CG by using generalized estimating equations with an exchangeable working correlations matrix to account for clustering within wards. We adjusted the analysis of the primary outcome for the binary stratification factor ward level exposure to wet work in the preceding year.

We performed a sensitivity analysis by using the observed (rather than predicted) ward level exposure to wet work and, for missing data, on the primary outcome at 12 months. Furthermore, a sensitivity analysis was carried out for participants with missing HECSI score data at 12 months. For this purpose, we performed three types of sensitivity analysis: (a) assuming the best possible outcome (HECSI score of 0); (b) assuming the worst possible outcome (highest observed HECSI score); and (c) applying multiple imputation by using baseline characteristics for the difference between baseline and 12‐month HECSI scores.

As some intervention studies have reported relative change in disease severity,^12^ we performed a post hoc analysis of the relative change in the HECSI score from baseline. This was calculated by use of the formula (ΔHECSI/HECSI T0) × HECSI T0. Furthermore, we used the MDC as proposed by Norman et al to a define meaningful improvement in HECSI score in this trial.^22^ The MDC was calculated as (0.5 × SD) = 0.5 × 10.2 = 5.1.

## 3 RESULTS

**Figure 1:**
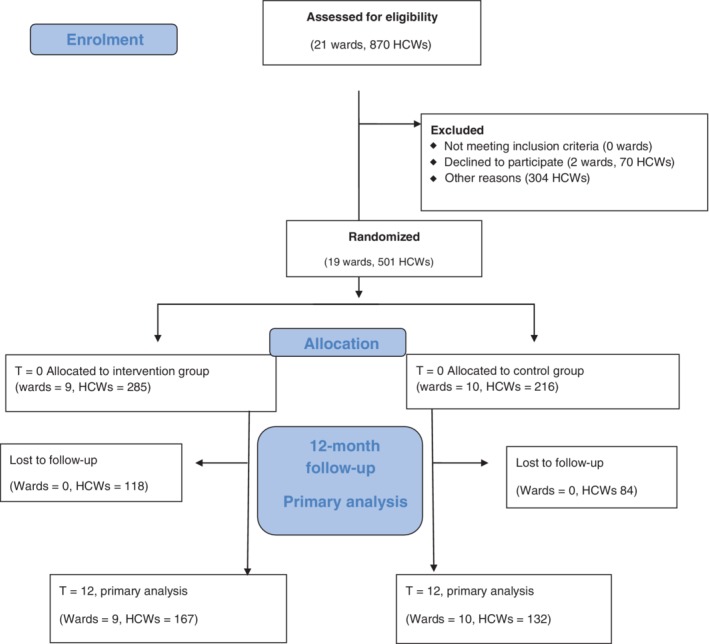
Flow diagram of wards and employees included in the Healthy Hands Project randomized controlled trial. HCW, heathcare worker

The study flowchart is shown in Figure 1. We included 501 HCWs from 19 wards; on average, 6 to 58 HCWs per ward were included. Nine wards with a total of 285 HCWs were allocated to the IG, and 10 wards with a total of 216 HCWs were allocated to the CG. Nine wards with a total of 169 HCWs in the IG and 10 wards with a total of 121 HCWs in the CG had high exposure to wet work (Table 1). All other wards and HCWs were classified as having low exposure to wet work. At the 12‐month follow‐up, all randomized wards remained in the study. However, 118 (41%) HCWs in the IG and 84 (39%) HCWs in the CG had been lost to follow‐up. The proportion of HCWs lost to follow‐up was similar in the two treatment groups (*P* = 0.65, ICC < 0.001). The per protocol population consisted of 299 participants (167 in the IG, and 132 in the CG).

**Table 1:** Baseline characteristics of the healthcare workers (HCWs) included in the trial

HCW characteristics	**Control group** **(10 wards, 216 HCWs)**	**Intervention group** **(9 wards, 285 HCWs)**
Types of ward	Ten clinical wards	Eight clinical wards, one outpatient clinic
High‐risk wards^a^	Five wards, 121 HCWs (56%)	Five wards, 169 HCWs (59%)
Sex, n (% male)	33 (15)	33 (12)
Working years, median (IQR) (n = 498)	11 (5‐20)	11 (5‐27)
Hours worked per week, mean (SD) (n = 499)	31 (7)	31 (6)
History of atopic dermatitis, n (%)	35 (16)	41 (14)
History of hand dermatitis in the past year n (%)	72 (33)	95 (33)
Frequency of use of hand alcohol, n (%) (n = 499)		
<5 times a shift	6 (3)	9 (3)
5‐10 times a shift	9 (4)	9 (3)
11‐15 times a shift	26 (12)	26 (9)
>15 times a shift	175 (81)	242 (85)
Frequency of hand washing, n (%) (n = 499)		
<5 times a shift	17 (8)	31 (11)
5‐10 times a shift	73 (34)	86 (30)
11‐15 times a shift	72 (33)	74 (26)
>15 times a shift	52 (24)	97 (34)
Frequency of glove use, n (%) (n = 497)		
<5 times a shift	30 (14)	20 (7)
5‐10 times a shift	48 (22)	34 (12)
11‐15 times a shift	56 (26)	60 (21)
>15 times a shift	82 (38)	171 (60)
Frequency of use of moisturizing creams before shift, n (%) (n = 497)		
Never	160 (74)	210 (74)
Approximately half of shifts	26 (12)	23 (8)
More than half of shifts	9 (4)	16 (6)
Almost always	22 (10)	34 (12)
Frequency of use of moisturizing creams during shift, n (%) (n = 497)		
Never	151 (70)	197 (69)
Approximately half of shifts	43 (20)	48 (17)
More than half of shifts	9 (4)	9 (3)
Almost always	15 (7)	13 (12)
Frequency of use of moisturizing creams after shift, n (%) (n = 497)		
Never	108 (50)	131 (46)
Approximately half of shifts	26 (12)	22 (13)
More than half of shifts	24 (11)	29 (10)
Almost always	58 (27)	88 (31)
Median HECSI score (IQR) (n = 501)	7 (4‐12)	8 (4‐13)
Mean NMF levels (SD) (n = 497)	4.6 (1.4)	4.3 (1.4)

Abbreviations: HECSI, Hand Eczema Severity Index; IQR, interquartile range; NMF, natural moisturizing factor.

a Risk estimate (high or low) based on soap exposure in the year before the trial.

The baseline characteristics of the HCWs are shown in Table 1. The proportion of males, number of hours worked per week, number of working years in a wet work occupation, proportion of HCWs with HD and a history of atopic dermatitis, and self‐reported use of soap, hand alcohol and hand creams were similar in the IG and the CG. The use of hand alcohol was high in both groups; 81% (405 HCWs) used it at least 15 times per shift. The majority of nurses (57%, 286 nurses) reported washing their hands at least 10 times per shift. High‐frequency glove use, that is, >15 times a shift, was significantly more common in the IG (59%, 167) than in the CG (36%, 77). Moreover, the largest proportion of nurses (70%, 351 nurses) reported that they “never” use creams before or during the shift.

At baseline, median HECSI scores were 8 (IQR 4‐13) in the IG and 7 (IQR 4‐12) in the CG. The numbers of HCWs with no, mild, moderate or severe symptoms according to HECSI score are shown in Table 2. Supplementary Table 1 shows each individual HD symptom separately scored by the HECSI index. Looking at the extremes, at follow‐up the increase in the proportion of HCWs reporting no symptoms was higher in the IG (15%) than in the CG (5%), whereas the decrease in the proportion of HCWs with moderate (16% in the IG vs 9% in the CG) and severe (6% in the IG vs 5% in the CG) HD was higher in the IG than in the CG. At baseline, the mean NMF levels were 4.3 (SD 1.4) in the IG and 4.6 (SD 1.4) in the CG (not significantly different).

**Table 2:** Classification of hand dermatitis severity in the intervention group (IG) and control group (CG) at baseline and follow‐up

	T = 0	T = 12	T = 0	T = 12
	IG (n = 285)	IG (n = 167)	CG (n = 216)	CG (n = 132)
No symptoms, n (%)	4 (1)	27 (16)	7 (3)	11 (8)
Mild, n (%)	199 (70)	129 (77)	151 (70)	104 (79)
Moderate, n (%)	63 (22)	10 (6)	45 (21)	16 (12)
Severe, n (%)	19 (7)	1 (1)	13 (6)	1 (1)
Median HECSI score (IQR)	8.0 (4.0‐13.0)	3.0 (1.0‐4.0)	7.0 (4.0‐12.0)	4.0 (2.0‐6.0)
Median NMF (IQR)	4.28 (3.41‐5.17)	3.2 7 (2.39‐4.12)	4.50 (3.57‐5.26)	3.30 (2.48‐4.19)

Abbreviations: HECSI, Hand Eczema Severity Index; IQR, interquartile range; NMF, natural moisturizing factor.

Mild: HECSI score of 1‐11.

Moderate: HECSI score of 12‐27.

Severe: HECSI score of ≥28.

### 3.1 Primary outcome: Absolute change in HECSI scores from baseline

The mean decreases in HECSI scores between baseline and 12‐month follow‐up (ΔHECSI) were −6.2 points (95%CI: −7.7 to −4.7) in the IG and − 4.2 (95%CI: −6.0 to −2.4) in the CG. The decrease in HECSI scores from baseline was significant in both groups (*P* < 0.0001). There was no significant difference in the change in HD severity between the IG and the CG; the decrease in HECSI scores was 2.6 points higher in the IG than in the CG, but this difference was not significantly different between groups (*P* = 0.17, ICC = 0.12). The linear model showed a significant effect (*P* = 0.04) of exposure to wet work.

The results from the sensitivity analysis using the best case (*P* = 0.20), worst case (*P* = 0.74) and multiple imputation (*P* = 0.09) were similar to those from the main analysis; that is, there was no difference in ΔHECSI between the IG and the CG. A sensitivity analysis using the current instead of historical data for wet work exposure (ie, purchase of soap during the trial period rather than data preceding the trial) still showed a significant effect of exposure (*P* = 0.02, ICC = 0.11). Nevertheless, there was no significant difference in ΔHECSI between the CG and the IG.

### 3.2 Post hoc analysis: Relative decrease in HECSI scores and MDC

We performed three post hoc analyses that had not been defined in the statistical analysis plan^19^: the relative change in HECSI scores, the MDC for HECSI scores, and subgroup analysis. The relative change in HECSI scores from baseline to follow‐up (percentage of the baseline value) is shown in Table 3. At follow‐up, the percentage changes in HECSI scores were 56% in the IG and 44% in the CG, representing a significant difference between the two arms (*P* = 0.001; ICC = 0.085). We calculated the MDC to be 5.1 HECSI points, which is a value that was only reached in the IG.

**Table 3:** Changes in Hand Eczema Severity Index (HECSI) score and natural moisturizing factor (NMF) levels from baseline to follow‐up in the intervention group (IG) and control group (CG)

	IG	CG	IG vs CG
ΔHECSI			
Mean (95%CI)	−6.2 (−7.7 to −4.7)	−4.2 (−6.0 to −2.4)	NS
Relative change (%)	56	44	*P* < 0.001^a^
ΔNMF			
Mean (SD)	−1.0 (1.6)	−1.2 (1.6)	NS

Abbreviations: CI, confidence interval; ΔHECSI, change in HECSI score from baseline to follow‐up; ΔNMF, change in NMF from baseline to follow‐up; NS, not significant.

a Difference in the change of the outcome from baseline between the IG and the CG.

To explore whether HD severity plays a role in the effectiveness of the intervention, we performed a subgroup analysis based on HECSI scores at baseline. To define the two subgroups, we used a cut‐off of HECSI score of 11 points, which was defined by Hald et al^21^ as mild dermatitis. The mean decreases in HECSI scores between baseline and 12‐month follow‐up (ΔHECSI) in the subgroup with mild HD (n = 124) were −3.0 points (95%CI: −3.5 to −2.5) in the IG and − 0.6 (95%CI: −1.3 to 0.24) in the CG (n = 95). In the subgroup with HECSI scores of >11, ΔHECSI values were −15.6 points (95%CI: −20.6 to −11) in the IG (n = 43) and − 13.4 (95%CI: −18.8 to −8.6) in the CG (n = 37). The results showed a significant effect of the intervention only in the subgroup with HECSI scores of ≤11, corresponding to early/mild HD (n = 219).

### 3.3 Secondary outcomes

At baseline, the NMF levels were similar between the IG and the CG. At follow‐up, NMF levels decreased in both groups: −1.0 (SD 1.6) in the IG, and −1.2 (SD 1.6) in the CG. There was no significant difference between the IG and the CG. The electronically measured cream use in the IG was, on average, 0.4 application events per HCW per shift during the trial.^28^

## 4 DISCUSSION

With respect to the primary and secondary outcomes, that is, respective changes in HECSI and NMF from baseline to follow‐up, this RCT could not provide evidence for effectiveness of the intervention.

### 4.1 Primary outcome

Although, in the present study, we found no significant effect of the intervention on the main outcomes, the intervention did have overall positive effects on the severity of HD symptoms, especially and significantly in specific subgroups. The decrease in HECSI score was larger in the IG than in the CG (respectively, 6.2 and 4.2 points). Furthermore, in contrast to the CG, the decrease in the IG exceeded the estimated MDC of 5.1 points. As some intervention studies have reported relative changes in disease severity,^12^ we performed a post hoc analysis, which showed relative improvement in HECSI score to be significantly larger in the IG than in the CG (56% vs 44%; *P* < 0.001).^12^ To explore whether HD severity plays a role in the effectiveness of the intervention, we performed a post hoc subgroup analysis. The results showed that the intervention did have a significant effect on HD severity in the subgroup of HCWs with mild HD (HECSI score of ≤11) (*P* < 0.0001), whereas, in the subgroup of HCWs with moderate to severe HD, there was no significant difference between the IG and the CG. This suggests that this intervention might be effective in preventing the progression of early skin barrier damage into clinical dermatitis, and may therefore be more suitable in primary prevention.

Intervention studies focusing on the effectiveness of skin care in high‐risk occupations are scarce, and skin care is often part of a multicomponent prevention programme.^12^ The effect of moisturizers vs no treatment has previously been investigated in three studies focused on the primary prevention of occupational HD; all three suggested a beneficial effect of moisturizers.^12^, ^13^, ^29^ On the basis of these three studies, a recent Cochrane review concluded that there may indeed be a clinically important risk reduction for the development of symptoms of HD (RR < 0.75, 95%CI: 0.46‐1.09; 507 participants), but emphasized that the quality of evidence is low.^30^

This intervention was aimed at reducing HD symptoms by improving skin care. Indeed, the intervention showed significantly higher cream use in the IG than in the CG.^28^ However, looking at the electronically collected data in the IG,^28^ the average frequency of 0.4 cream applications per nurse per shift was much lower than recommended in the present study and in current guidelines (two applications per shift).

In this trial, we provided, both in the IG and in the CG, basic educational lessons to achieve the same level of knowledge, and tried to avoid differences between the two arms. As previously studied, education itself could have a positive effect on the severity of HD.^2^, ^31^ This may at least partly explain why skin symptoms were improved not only in the IG but also (although to a lesser extent) in the CG during the trial.

### 4.2 Secondary outcome

Interestingly, NMF levels in both groups were significantly decreased at follow‐up as compared with baseline. Exposure to water and soap has been shown to reduce NMF levels by different mechanisms^32^, ^33^, ^34^, ^35^; however, this cannot explain the decrease in NMF levels, as wet work exposure did not change during the trial. One of the possible explanations might be the introduction of a new disinfectant, containing n‐propanol instead of the formerly used ethanol, shortly before the start of our trial. In addition to the stronger skin barrier‐damaging effect of n‐propanol than of ethanol,^36^ our recent work also showed an NMF level‐decreasing effect of n‐propanol.^33^ The introduction of this new disinfectant might also explain another discrepancy in the NMF results. A reduction in NMF level has been suggested to be a contributory factor for dry skin in HD patients, and one may expect that a decrease in NMF level would parallel an increase in skin symptoms; however, this was not the case in the present study. The new disinfectant formula contained glycerol, which is a frequently used humectant in skin care products.^37^ Notably, glycerol was also present in the hand creams provided to the IG. It may be speculated that the addition of glycerol to the new disinfectants could have at least partly compensated for the NMF level‐decreasing effect on skin hydration associated with propanol. These positive effects of glycerol might also have interfered with the primary outcome, and may explain why the HECSI score was also improved in the CG at follow‐up.

### 4.3 Strengths and limitations

The strengths of our study included stratified randomization, controlling for cross‐contamination, and comparable groups with regard to baseline values of the primary and secondary outcomes and covariates. In contrast to studies focused on patients, we included one high‐risk occupational group (HCWs) under real occupational conditions. Assessment of outcomes and statistical analysis were performed blinded to allocation. To the best of our knowledge, this is the first study to use real‐time monitoring to quantitatively assess the consumption of creams. In addition, we applied an objective and validated tool (HECSI score) for clinical assessment of the severity of HD by a trained physician. A quantitative biomarker of early skin barrier damage (NMF) was included as our secondary outcome.

There are several factors that might explain the lack of effect in the present RCT. One of the main drawbacks of this trial is the substantial loss of power resulting from overestimation of the number of available clusters, a higher loss to follow‐up, and higher ICC values than assumed. Driven by organizational constraints during recruitment, we had to regard several subdepartments together as one cluster. Although these wards were at different locations, they shared the same management, and cross‐contamination was likely, as HCWs rotated within such large clusters. However, this aspect was not foreseen when power was calculated, leading to a considerable loss of power (post hoc power of 56%) because of the lower number of clusters that could be included. The included number of HCWs, however, was very close to the planned sample size.

At the individual level, there was considerable loss to follow‐up, which was similar in both groups (41% in the IG and 39% in CG). We did not systematically record the reasons for dropout, but, from informal discussions with the supervisors, we found that the most commonly reported reasons for loss to follow‐up were change of ward or job during the trial period, sick leave, maternity leave, annual leave during the final measurement period at follow‐up, or simply no desire to continue to participate. The sensitivity analysis showed that loss to follow‐up was independent of severity of hand symptoms, and therefore the risk of attrition is not likely.

This trial was not designed to exclude atopic dermatitis of the hands, allergic contact dermatitis, or non‐occupational hand dermatitis (eg, attributable to wet work activities in leisure time). It is known that there are several types of occupational HD, which overlap and are difficult to distinguish.^38^ Moreover, the mild symptoms of HD (mostly only slight erythema) observed in most HCWs at entry, implying that there was limited room for improvement, constitute another factor that might have diluted the effect of the intervention with regard to severity. As our study population was a working population, HECSI scores were markedly lower than in patients in secondary prevention studies.^21^ According to the classification proposed by Hald et al,^21^ our study population was considered to represent mainly mild dermatitis (HECSI score of <11). There is no clear lower threshold value for HECSI score to define HD, as there is no clear agreement on when irritant skin changes are defined as HD. As emphasized by Bauer et al^30^ in a recent Cochrane study, future research should be directed towards further developing and validating HD scoring systems for better and standardized discrimination of irritant skin changes from ICD.^30^ Finally, the change in hand alcohol (from an ethanol‐containing to a propanol‐containing formula and the addition of glycerol) on the wards just before this trial started is also considered to be a limitation. This is relevant, as it may have influenced both primary and secondary outcomes.

## 5 CONCLUSION

This is the first trial to report the effectiveness of a prevention programme in the healthcare environment focused on the application of creams combined with continuous monitoring and feedback on skin care performance. This intervention was reported to improve hand cream use.^28^ However, the IG did not show significantly better improvement in the primary outcome (ΔHECSI) and secondary outcome (ΔNMF) than the CG. Notwithstanding this, the intervention showed overall positive effects on the severity of HD symptoms, supporting the benefits of creams in the workplace, in particular in HCWs with mild HD. As occupational health interventions tend to be complex and dependent on context, evaluation based strictly on the primary and secondary outcomes in the total group might not reflect the overall benefit of the intervention.^39^ In the present study, we did not focus on the barriers to and facilitators of hand cream use; however, the fact that cream use, despite it resulting in some improvement during the trial, still remains quite low, is intriguing. To design successful HD prevention strategies in the future, further investigation of these factors is needed.

## CONFLICTS OF INTEREST

The PhD project of M. Soltanipoor was funded by an unrestricted grant from DEB Group Ltd, Denby, UK. DEB Group Ltd also funded the dispensers with study creams. The participants in the study were covered by their work insurance. The sponsor was not involved in the study design, execution of the trial, or writing study reports. The authors report no further conflicts of interest.

## Supporting information


**Table S1.** Symptoms of hand dermatitis in the intervention group (IG) and the control group (CG) at baseline and follow‐up
