# Supplementary material for: Effectiveness of a skin care programme for the prevention of contact dermatitis in healthcare workers (the Healthy Hands Project): A single‐centre, cluster randomized controlled trial
Source: Contact Dermatitis. 2019 Mar 15;80(6):365–73. doi: 10.1111/cod.13214 (PMC6593800; doi:10.1111/cod.13214)
Supplement: Supplementary file 1 — Table S1. Symptoms of hand dermatitis in the intervention group (IG) and the control group (CG) at baseline and follow‐up [file COD-80-365-s001.docx]

**Supplementary Table 1. Symptoms of hand dermatitis in the intervention group (IG) and control group (CG) at baseline and follow-up**

|  | T0 | T12 | T0 | T12 |
| --- | --- | --- | --- | --- |
| *Symptoms* | **IG (n=285)** | **IG (n=167)** | **CG (n=216)** | **CG (n=132)** |
| Erythema | 261 (92%) | 98 (58%) | 200 (93%) | 111 (84%) |
| Infiltration | 31 (11%) | 9 (5%) | 19 (9%) | 6 (5%) |
| Vesicles | 9 (3%) | 1 (1%) | 18 (8%) | 4 (3%) |
| Fissures | 94 (33%) | 23 (14%) | 81 (38%) | 55 (42%) |
| Scaling | 141 (50%) | 68 (41%) | 123 (57%) | 100 (76%) |
| Oedema | 1 (1%) | 1 (1%) | 4 (2%) | 0% (3%) |
